# Analysis of the Transcriptional Dynamics of Regulatory Genes During Peanut Pod Development Caused by Darkness and Mechanical Stress

**DOI:** 10.3389/fpls.2022.904162

**Published:** 2022-05-26

**Authors:** Yuanyuan Cui, Jianxin Bian, Yuying Lv, Jihua Li, Xing Wang Deng, Xiaoqin Liu

**Affiliations:** ^1^Shandong Laboratory for Advanced Agricultural Sciences at Weifang, Peking University Institute of Advanced Agricultural Science, Weifang, China; ^2^School of Advanced Agricultural Sciences, Peking University, Beijing, China

**Keywords:** peanut pod development, transcriptome, stasis phenomenon, light, dark, mechanical stress

## Abstract

Peanut is an oil crop with important economic value that is widely cultivated around the world. It blooms on the ground but bears fruit underground. When the peg penetrates the ground, it enters a dark environment, is subjected to mechanical stress from the soil, and develops into a normal pod. When a newly developed pod emerges from the soil, it turns green and stops growing. It has been reported that both darkness and mechanical stress are necessary for normal pod development. In this study, we investigated changes in gene expression during the reverse process of peg penetration: developmental arrest caused by pod (Pattee 3 pods) excavation. Bagging the aerial pods was used to simulate loss of mechanical pressure, while direct exposure of the aerial pods was used to simulate loss of both mechanical pressure and darkness. After the loss of mechanical stress and darkness, the DEGs were significantly enriched in photosynthesis, photosynthesis–antenna proteins, plant–pathogen interaction, DNA replication, and circadian rhythm pathways. The DNA replication pathway was enriched by down-regulated genes, and the other four pathways were enriched by upregulated genes. Upregulated genes were also significantly enriched in protein ubiquitination and calmodulin-related genes, highlighting the important role of ubiquitination and calcium signaling in pod development. Further analysis of DEGs showed that *phytochrome A* (*Phy A*), *auxin response factor 9* (*IAA9*), and *mechanosensitive ion channel protein* played important roles in geocarpy. The expression of these two genes increased in subterranean pods but decreased in aerial pods. Based on a large number of chloroplast-related genes, calmodulin, kinases, and ubiquitin-related proteins identified in this study, we propose two possible signal transduction pathways involved in peanut geocarpy, namely, one begins in chloroplasts and signals down through phosphorylation, and the other begins during abiotic stress and signals down through calcium signaling, phosphorylation, and ubiquitination. Our study provides valuable information about putative regulatory genes for peanut pod development and contributes to a better understanding of the biological phenomenon of geocarpy.

## Introduction

Peanuts are an annual herb belonging to the legume family and are cultivated around the world. Its embryos are an important source of protein for humans (Wang et al., 2021). As an active-geocarpy crop, peanuts have a typical fruiting mechanism. They first blossom aboveground. After fertilization, the ovary stalks (known as pegs) grow underground and push the ovary into the ground, where the fruit develops until it reaches maturity (Tan et al., 2010). Failure to penetrate leads to seed abortion, which affects peanut production. For many years, the characteristic pod development of peanuts has piqued the interest of botanists (Kumar et al., 2019).

Previous studies have demonstrated that light enhances gynophore elongation but inhibits pod formation *in vitro* (Ziv and Zamski, 1975). Both darkness and mechanical stress are important factors involved in pod formation after penetration (Zamski and Ziv, 1976). By perceiving changes in their external environment, gynophores regulate multiple endogenous hormones and promote the enlargement of the ovaries and pod development (Kumar et al., 2019). Hormone measurements before and after penetration showed that the amount of ethylene released by the gynophore after penetration was twice as much as that of the aerial gynophore (Shlamovitz et al., 1995). Both IAA (indoleacetic acid) and ABA (abscisic acid) decreased after gynophore penetration, and gibberellic acid [GA_1_, GA_3_, GA_8_, and 6-deoxoCS (castasterone)] decreased after penetration, and then increased as the pod expanded (Li, 2020). Tinfoil wrapping and pinching pods experiment also demonstrated that GA, ABA, and BR decreased under dark conditions and stimulated treatment of aerial gynophores (Li et al., 2014). Therefore, the development of subterranean pods was accompanied by an increase in ethylene and a decrease in four plant hormones, including IAA, ABA, GA, and BR. Kumar et al. (2019) hypothesized that gynophores could maintain high ABA levels during light exposure to prevent embryo growth until penetration, after which dark could inhibit ABA levels and allow embryo growth to resume.

Several omics methods were used to analyze geocarpy in peanuts. Hundreds of transcripts associated with gravitropism and photomorphogenesis and proteins are involved in the gravity response, light and mechanical stress, hormone biosynthesis, and transport and are associated with geocarpy in peanuts (Zhao et al., 2015; Chen et al., 2016). Additionally, genes involved in the ubiquitin-proteasome system (UPS) and cell wall modification could function as candidates to regulate peanut pod development (Zhu et al., 2013, 2014). Comparative transcriptome analysis demonstrated that genes related to hormone metabolism, signaling, photosynthesis, light signaling, cell division and growth, carbon and nitrogen metabolism, and stress responses were highlighted after gynophore penetration (Xia et al., 2013). Genes involved in light signaling transduction, such as *PHYA* (*phytochrome A*), *CRY2* (*cryptochromes 2*), and *CRY3* (*cryptochromes 3*), had low expression during pod development. The *COP1 interacting protein 7* (*CIP7*) decreased when gynophores just penetrated the soil and then increased as the pod expanded underground (Liu et al., 2020).

Peanut seed abortion caused by peg penetration failure is another popular research topic. Comparative proteome analysis between aerial (including seed abortion samples) and subterranean pods showed that differentially abundant proteins (DAPs) were primarily involved in photosynthesis, oxidative stress response, lignin synthesis, and fatty acid biosynthesis (Zhu et al., 2013). Transcriptome analysis of aerial (including seed abortion samples) and subterranean pods development demonstrated that differentially expressed genes (DEGs) primarily participated in the biological processes of hormone response, cell apoptosis, embryonic development, and light signaling. HPLC analysis of aerial (seed abortion) and subterranean pods showed that IAA content significantly increased in aerial pods rather than subterranean pods, highlighting the important role of IAA in seed abortion of aerial pods (Zhu et al., 2014). In addition, two senescence-associated genes were identified as significantly upregulated in the aerial pod, which could also contribute to embryo abortion in aerial pods (Chen et al., 2013).

There is a common phenomenon: gynophores that have been buried and expanded stopped developing when exposed to light on the ground (Pan, 1982), and it is unclear whether the absence of darkness and mechanical stress prevent further pod development. This study experimentally simulated this phenomenon by digging out the enlarged pods that penetrated the soil and subjecting them to conditions including the loss of mechanical stress and the loss of both mechanical stress and darkness. The gene expression in different states was then examined by transcriptome analysis, the results of which contribute to a better understanding of the molecular mechanism regulating peanut pod development.

## Materials and Methods

### Plant Materials

The peanut cultivar Haihua 1 (Zhang, 1986) was used as the experimental material and was planted at the Experimental Station of the Institute of Advanced Agricultural Sciences of Peking University. Peanut pod shells expand earlier than the seeds after penetrating the soil. The tissue first senses and adapts to the underground environment; as a protective and sensing organ, it ensures subsequent seed expansion. Since changes in the external environment are first perceived by the shell, the shell was the processed sample unless otherwise specified.

### Sample Preparation

Pattee 3 pods (Pattee et al., 1974), which have small, undeveloped seeds, we retrieved approximately 10–15 days after penetration into the soil and named the sample DM (dark and mechanical stress). This was used as the control (Supplementary Figure 1). The pods were immediately wrapped in a breathable black paper bag to simulate losing only mechanical stress and were named sample D (dark). Given that the seeds of the aerial pods turned green after 3 days of illumination, sunset on the third day was considered the final sampling point. Two sampling points were added (sunset on the first and second day), for a total of three sampling points. Therefore, the D samples harvested after 10 (day 1), 34 (day 2), and 58 (day 3) h of treatment were named D1, D2, and D3, respectively. Next, the pods were exposed to air to simulate losing both darkness and mechanical stress and were named sample L (Light). The L samples were harvested after 10 (day 1), 34 (day 2), and 58 (day 3) h of treatment and were named L1, L2, and L3, respectively (Supplementary Figure 1b). The pod shells of the sample DM, D1, D2, D3, L1, L2, L3, and the seed of sample D3 (named D3S) and L3 (named L3S) were isolated and stored at −80°C. Each sample had 15 pods, and three biological replicates were performed for each sample.

### RNA-Seq

RNA was isolated from finely ground samples using a Takara Mini-BEST Plant RNA Extraction Kit (Takara, Dalian, China). The total amounts and integrity of RNA were measured by an Agilent 2100 Bioanalyzer. Total RNA was used as input material to construct the cDNA libraries with an NEB Next^®^ Ultra™ RNA Library Prep Kit for Illumina ^®^ (NEB, Ipswich, MA, United States). The Illumina NovaSeq 6000 was used for paired-end sequencing (2 × 150 bp) to construct libraries, and the image data was then transformed into sequence data by CASAVA base recognition. The raw data of fastq format were first processed to filter out low-quality sequences. All downstream analyses were based on clean, high-quality data. The raw data obtained from this experiment has been deposited in NCBI (SRA accession: PRJNA770556).

High-quality sequences were matched with the reference genome of Tifrunner (Bertioli et al., 2019) using Hisat2 (v2.0.5). Documentation related to gene annotation was downloaded from the website https://v1.legumefederation.org/data/v2/Arachis/hypogaea/genomes/Tifrunner.gnm2.J5K5. DEG, GO (Gene Ontology), and KEGG (Kyoto Encyclopedia of Genes and Genomes) were analyzed according to a previously described method (Cui et al., 2021). Gene expression was visualized using TBtools (Chen et al., 2020).

Genes with FPKM greater than 5 in 50% of the samples were selected for Weighted Gene Co-Expression Network Analysis (WGCNA) (Langfelder and Horvath, 2008). Genes were then filtered by median absolute deviation (MAD) since low-expressed or non-varying genes usually represented noise. A soft threshold power of 26, a minimum module size of 60, and a merge cut height of 0.30 were used for module construction. The eigengene was calculated for each module and was then used to search for associations with mechanical stress and darkness. Since mechanical stress and darkness were not quantitative traits, they were considered to be present (1) or absent (0). Gene significance (GS) and module membership (MM) was a priority to screen for important genes within the module. Finally, the networks were visualized using Cytoscape (Kohl et al., 2011).

### Quantitative Real-Time Polymerase Chain Reaction

RNA extraction was performed according to the same methods as RNA-Seq. A Prime Script RT Reagent Kit (Takara, Dalian, China) was used to synthesize the cDNA of each sample. Gene-specific primers were designed by Primer3^1^ and the *elongation factor 1B* (*Arahy.E3HYWR*) was used as an internal control (Supplementary Table 1). qPCR was performed on the ABI 7500 Fast using three replicates and TB Green^®^ Premix Ex TaqTM (Takara, Dalian, China). The reaction conditions were: 95°C for 5 min, 40 thermal cycles of 95°C for the 30 s, then 60°C for 30 s. The 2−△△CT method was used for relative quantification analysis (Livak and Schmittgen, 2001). Significance analysis was performed using SPSS 26.0 software.

## Results

### Overall Gene Expression Changes Caused by Darkness and Mechanical Stress Loss

A total of 72,588 unigenes were annotated from the transcriptome data (9 samples), including 5,583 novel genes (Supplementary Table 2). Sample correlation analysis showed that 9 samples could be clustered into three categories (Supplementary Figure 2). Two seed samples were clustered together, and the DM sample itself was a category. Three pod shell D samples and three pod shell L samples were clustered together, and the six samples comprised a category. Although the D and L samples were treated differently, their gene expression was relatively similar. This differed from the untreated DM samples (Supplementary Figure 2).

### Enrichment Analysis of Differentially Expressed Genes After the Loss of Mechanical Stress

**FIGURE 1:**
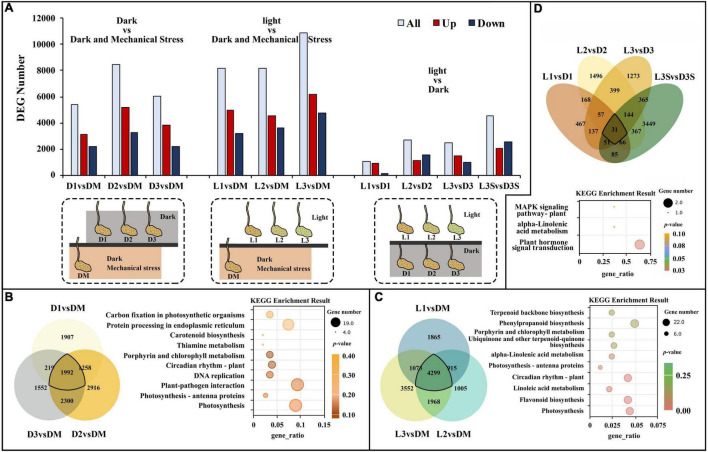
DEGs and significant KEGG pathways for DEGs in D vs. DM, L vs. DM, and L vs. D. **(A)** The numbers of DEGs in D vs. DM, L vs. DM, and L vs. D; the treatment diagram is shown at the bottom of the bar chart and the specific materials and treatment are shown in Supplementary Figure 1b; **(B)** significant KEGG pathways for DEGs in D vs. DM; **(C)** significant KEGG pathways for DEGs in L vs. DM; **(D)** significant KEGG pathways for DEGs in L vs. D. The “gene_ratio” means the ratio of the number of DEGs annotated in the KEGG pathway to the total number of DEGs that can be annotated in the KEGG database.

Differentially expressed gene analysis demonstrated that there were 5,376, 8,436, and 6,063 DEGs in D1 vs. DM, D2 vs. DM, and D3 vs. DM, respectively. These were caused by a loss of mechanical stress. These three comparisons shared 1,992 DEGs (Figures 1A,B). The significant GO enrichment term for these DEGs was primarily thylakoid, photosynthetic membrane, and oxidoreductase complex (Supplementary Table 3). The top 5 KEGG enrichment terms for these DEGs were photosynthesis, photosynthesis–antenna proteins, plant–pathogen interaction, DNA replication, and circadian rhythm (Figure 1B).

Further analysis of upregulated and downregulated DEGs demonstrated that upregulated DEGs were mainly primarily in photosynthesis, protein ubiquitination, flavonoid biosynthesis, and circadian rhythm. However, downregulated DEGs were mainly enriched in DNA replication, protein folding, and ribosome (Table 1). Without soil protection, the mechanical stress in the pod shell is lost, and genes related to photo stress are significantly upregulated. This modifies or degrades some proteins through ubiquitination, which slows down life activities.

**TABLE 1:** GO and KEGG enrichment analysis of DEGs in D vs. DM comparisons.

Description		Upregulation	Downregulation
GO	D1 vs. DM	Photosynthesis; Thylakoid; Thylakoid part	Protein folding; DNA replication; Response to endogenous stimulus
	D2 vs. DM	Protein ubiquitination; Protein modification by small protein conjugation; Photosynthetic membrane	DNA replication; DNA metabolic process; Protein folding
	D3 vs. DM	Photosynthesis; Cell communication; Protein ubiquitination	Translation; Peptide biosynthetic process; Peptide metabolic process
KEGG	D1 vs. DM	Photosynthesis; Linoleic acid metabolism; Alpha-Linolenic acid metabolism	Protein processing in endoplasmic reticulum; Ribosome biogenesis in eukaryotes; DNA replication
	D2 vs. DM	Photosynthesis; Flavonoid biosynthesis; Plant-pathogen interaction	DNA replication; Phagosome
	D3 vs. DM	Photosynthesis; Circadian rhythm – plant; Plant-pathogen interaction	Ribosome; DNA replication

*Only the first three significantly (p-adjust < 0.05) enriched terms/pathways (the most significant one is underlined) are listed here, others can be found in Supplementary Table 4.*

### Enrichment Analysis of Differentially Expressed Genes After the Loss of Mechanical Stress and Darkness

Darkness and mechanical pressure are lost when the pod is illuminated. There were 8,152, 8,187, and 10,829 DEGs in L1 vs. DM, L2 vs. DM, and L3 vs. DM, respectively. These three comparisons shared 4,299 DEGs, which was 2.16 times as many DEGs than were caused by the loss of mechanical stress (Figures 1A,C). The significant GO enrichment term for these DEGs was mainly photosynthesis, isoprenoid biosynthetic and metabolic processes, and terpenoid biosynthetic and metabolic processes (Supplementary Table 3). The top five KEGG enrichment terms for these DEGs were photosynthesis, flavonoid biosynthesis, linoleic acid metabolism, circadian rhythm, and photosynthesis–antenna proteins (Figure 1C).

Gene ontology enrichment of upregulated DEGs in L vs. DM showed similar terms to D vs. DM: photosynthesis and protein ubiquitination. Isoprenoid biosynthetic and metabolic processes were also the significant GO terms in L vs. DM but not in D vs. DM (Table 2 and Supplementary Table 4). KEGG enrichment of upregulated DEGs in L vs. DM showed that they were primarily enriched in flavonoid biosynthesis and circadian rhythm. Downregulated DEGs were significantly enriched in plant hormone signal transduction, except that they were enriched in DNA replication and ribosome as in D vs. DM (Table 2). The loss of both darkness and mechanical stress produced the same gene expression changes that occurred as loss of mechanical stress, plant hormone, and more secondary metabolites (isoprenoid, terpenoid, flavonoid, and phenylpropanoid)-related gene expression changes (Figure 1 and Table 2).

**TABLE 2:** GO and KEGG enrichment analysis of DEGs in L vs. DM comparisons.

Description		Upregulation	Downregulation
GO	L1 vs. DM	Photosynthesis; Multi-organism process; Isoprenoid metabolic process	DNA replication; DNA metabolic process; Response to endogenous stimulus
	L2 vs. DM	Photosynthesis; Isoprenoid metabolic process; Isoprenoid biosynthetic process	Movement of cell or subcellular component; Microtubule-based movement; DNA replication
	L3 vs. DM	Photosynthesis; Protein ubiquitination; Protein modification by small protein conjugation	Movement of cell or subcellular component; Microtubule-based movement; Microtubule-based process
KEGG	L1vs. DM	Flavonoid biosynthesis; Photosynthesis; Circadian rhythm–plant	Plant hormone signal transduction
	L2 vs. DM	Photosynthesis; Flavonoid biosynthesis; Phenylpropanoid biosynthesis	Phagosome; DNA replication
	L3 vs. DM	Flavonoid biosynthesis; Photosynthesis; Circadian rhythm–plant	Ribosome

*Only the first three significantly (p-adjust < 0.05) enriched terms/pathways (the most significant one is underlined) are listed here, others can be found in Supplementary Table 5.*

### Enrichment Analysis of Differentially Expressed Genes Caused by Light Exposure

The difference between the D and L samples was exposure. Green pericarps and seed coats were observed in L samples on day 3 (after 58 h) but not in the unexposed D samples (Supplementary Figure 1). Considering that seeds in the D3 and L3 samples were different, they (D3S and L3S) were also included in the gene expression analysis to provide information for seed developmental arrest caused by light stress.

The number of DEGs in the D and L samples decreased significantly compared with the D vs. DM and L vs. DM samples (Figures 1A,D). There were 1,062, 2,728, 2,457, and 4,558 DEGs in L1 vs. D1, L2 vs. D2, L3 vs. D3, and L3S vs. D3S, respectively. These four comparisons shared only 31 DEGs (Figure 1D). The significant GO enrichment term for these DEGs was mainly related to protein ubiquitination, which enriched two *U-box domain-containing protein/E3 ubiquitin ligases* (*J6YH7R* and *CT4JMD*). KEGG analysis showed that one *EBF* (*EIN3-binding F-box protein*) gene (*Y2W1T6*) was enriched in plant hormone signal transduction and the MAPK signaling of the KEGG pathway. Their expression in pod shells significantly increased out of the soil, and their expression was higher in exposed seeds than in unexposed seeds (Figure 1D, Supplementary Table 3, and Supplementary Figure 3).

According to GO analysis, compared with shaded pod shells, upregulated genes in the exposed pod shells were primarily enriched in photosynthesis and oxidative stress; downregulated genes were primarily enriched in carbohydrate metabolism and signal transduction processes. On the other hand, KEGG analysis demonstrated that the upregulated genes in exposed pod shells were primarily enriched in secondary metabolism pathways (such as flavonoid biosynthesis and phenylpropanoid biosynthesis) and circadian rhythm. Downregulated genes were primarily enriched in plant–pathogen interactions and plant hormone signal transduction (Table 3).

**TABLE 3:** GO and KEGG enrichment analysis of DEGs in L vs. D comparisons.

Description		Upregulation	Downregulation
GO	L1 vs. D1	Response to oxidative stress; Multi organism process; Cell recognition	Cellular carbohydrate metabolic process; Polysaccharide catabolic process; Inositol metabolic process
	L2 vs. D2	Photosynthesis; Response to oxidative stress; Photosynthesis, light reaction	Phosphorelay signal transduction system; Calmodulin binding; Carbohydrate binding
	L3 vs. D3	Photosystem I reaction center; Photosystem I; Photosynthetic membrane	Defense response; Response to biotic stimulus; Inositol metabolic process
	L3S vs. D3S	Organic acid catabolic process; Carboxylic acid catabolic process; Fatty acid metabolic process	Movement of cell or subcellular component; Microtubule-based movement; Microtubule-based process
KEGG	L1 vs. D1	Flavonoid biosynthesis; Circadian rhythm–plant; Plant-pathogen interaction	
	L2 vs. D2	Photosynthesis; Phenylpropanoid biosynthesis; Carotenoid biosynthesis	Plant-pathogen interaction; Circadian rhythm–plant
	L3 vs. D3	Flavonoid biosynthesis; Circadian rhythm–plant; Phenylpropanoid biosynthesis	Plant hormone signal transduction
	L3S vs. D3S	Plant-pathogen interaction; Plant hormone signal transduction; Alpha-Linolenic acid metabolism	Glycolysis/Gluconeogenesis; Starch and sucrose metabolism; Carbon metabolism

*Only the first three significantly (p-adjust < 0.05) enriched terms/pathways (the most significant one is underlined) are listed here, others can be found in Supplementary Table 6.*

Differentially expressed gene enrichment results in pod shells and seeds under the two conditions (dark and light) were different. The upregulated genes in seeds were mainly enriched in the organic acid catabolic process (GO term) and plant–pathogen interaction (KEGG pathway), while downregulated genes in seeds were primarily enriched in the movement of a cell or subcellular component (GO term) and glycolysis/gluconeogenesis (KEGG pathway) (Table 3).

### Differentially Expressed Genes Shared by D vs. DM and L vs. DM (Caused by Loss of Mechanical Stress)

**FIGURE 2:**
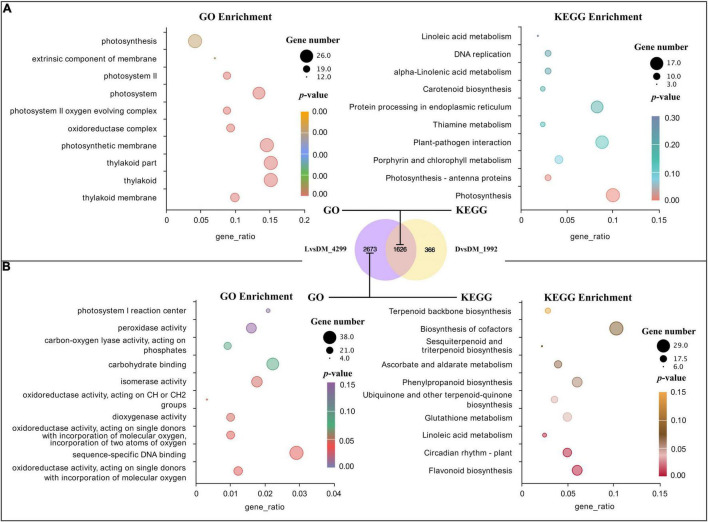
GO and KEGG analysis for DEGs shared by D vs. DM and L vs. DM and found only in L vs. DM. **(A)** Significant GO terms and KEGG pathways for DEGs shared by D vs. DM and L vs. DM; **(B)** significant GO terms and KEGG pathways for DEGs found only in L vs. DM. The “gene_ratio” means the ratio of the number of DEGs annotated in the KEGG pathway to the total number of DEGs that can be annotated in the KEGG database.

The common feature of the D and L samples was the loss of mechanical stress, indicating that this was the cause of the shared 1,626 DEGs in D vs. DM (1,992 DEGs) and L vs. DM (4,299 DEGs). Interestingly, the GO and KEGG enrichment term of these 1,626 DEGs was mainly related to the photosystem (Figure 2A). This could be because there was a connection between mechanical stress and light or because a small amount of light could penetrate the bag and cause significant changes in gene expression.

### Differentially Expressed Genes Found Only in the L vs. DM (Primarily Responsive to Light)

In addition to the loss of mechanical stress, the L sample also lost dark conditions. Therefore, DEGs found only in the L vs. DM (2,673 DEGs) could be primarily responsive to light. These significantly enriched GO terms of these DEGs were mainly related to oxidoreductase activity. The largest number of DEGs were enriched in GO in sequence-specific DNA binding. The significantly enriched pathways were secondary metabolism-related, and the most significant was flavonoid biosynthesis (enriched 17 DEGs). The largest number of DEGs (29) were enriched in the biosynthesis pathway of cofactors (Figure 2B). The expression of genes related to oxidoreduction, secondary metabolism, and circadian rhythm increased in illuminated pod shells, which is consistent with the previous enrichment analysis results of L vs. DM and L vs. D (Tables 2, 3).

### Differentially Expressed Genes Related to Circadian Rhythm

**FIGURE 3:**
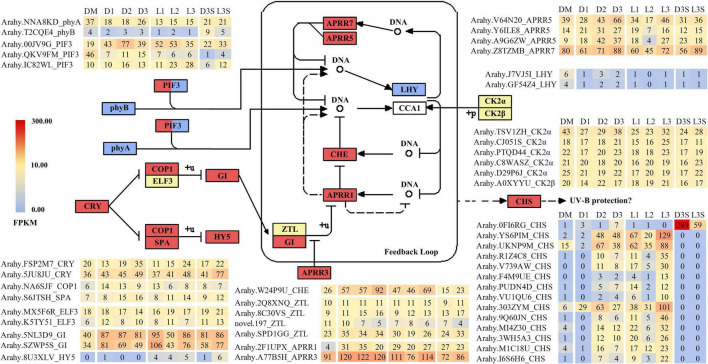
DEGs related to circadian rhythm. Only those with FPKM value ≥ 10 in at least one sample are listed; the others can be found in Supplementary Table 7.

Previous research has demonstrated that the expression of many genes involved in plant circadian rhythms changes during ped penetration (Xia et al., 2013; Clevenger et al., 2016; Zhang et al., 2016; Liu et al., 2020). Changes in circadian rhythm-related genes were also observed during the reverse process of penetration (Tables 1–3). When darkness and mechanical stress were lost, most circadian rhythm-related DEGs were upregulated in response to environmental changes, including *CRY* (*cryptochrome*), *COP1* (*constitutive photomorphogenic 1*), *SPA* (*suppressor of phyA*), and *HY5* (*elongated hypocotyl 5*) (Figure 3). However, *phyA* (*phytochrome A*), *phyB* (*phytochrome B*), and two *LHY* (*late elongated hypocotyl*) were all downregulated during this process. Interestingly, there were many significantly upregulated *CHS* (*chalcone synthase*) identified during this process, except for *OFI6RG*, which seemed to be primarily expressed in seeds and downregulated by 4.90 times in response to light.

### Differentially Expressed Genes Related to Protein Ubiquitination and Calmodulin

In both the D vs. DM and L vs. DM comparisons, the upregulated DEGs were enriched in protein ubiquitination and calcium ion binding (Tables 1, 2; Supplementary Tables 3, 4). Ubiquitination plays an important role in photomorphogenesis and is involved in regulating cell cycles, proliferation, gene expression, transcription regulation, signaling, and almost all other life activities (Han et al., 2020). In addition, calmodulin is an important messenger molecule in plant life (Zielinski, 1998). Therefore, we screened for the upregulation of ubiquitination and calcium ion binding-related genes after the loss of mechanical stress and darkness, which could play an important role in signal transduction.

**FIGURE 4:**
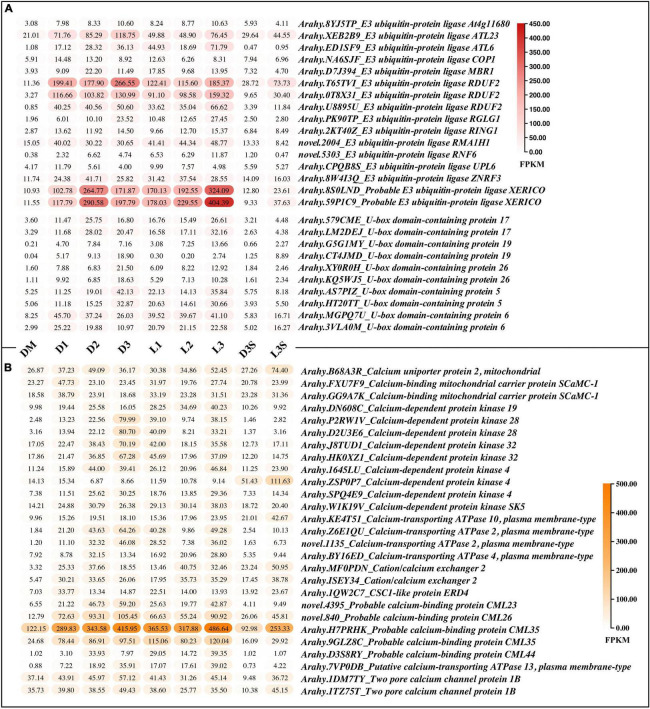
DEGs related to protein ubiquitination and calcium ion binding. **(A)** DEGs related to protein ubiquitination; **(B)** DEGs related to calmodulin, only those with FPKM value ≥ 30 in at least one sample are listed; the others can be found in Supplementary Table 8.

There were two main kinds of ubiquitination-related genes that were upregulated after the loss of mechanical stress and darkness: *E3 ubiquitin-protein ligase* and *U-box domain-containing protein* (Figure 4A). Four *E3 ubiquitin-protein ligases* (RDUF2:*T65TVI* and *0T8* × *31*; XERICO: *8S0LND* and *59P1C9*) increased by more than 10 times in pod shells after leaving the soil. In *Arabidopsis*, E3 ubiquitin-protein ligase RDUF2 is involved in positively regulating abscisic acid-dependent drought stress responses (Kim et al., 2012). Overexpressing E3 ubiquitin-protein ligase XERICO in *Arabidopsis* confers drought tolerance through increased abscisic acid biosynthesis (Ko et al., 2006).

A total of 27 calcium ion binding related genes, with FPKM greater than 30 in at least one sample, were upregulated, including *calcium-binding protein*, *calcium-dependent protein kinase*, *calcium-transporting ATPase*, *two pore calcium channel protein*, and *cation/calcium exchanger* (Figure 4B). The *calcium-binding protein* (*Arahy.H7PRHK*) had the highest average expression, and the *calcium-dependent protein kinase 4* (*Arahy.ZSP0P7*), which was more highly expressed in seeds, was upregulated in response to light.

### Differentially Expressed Genes Related to Plant Hormone Signal Transduction

**FIGURE 5:**
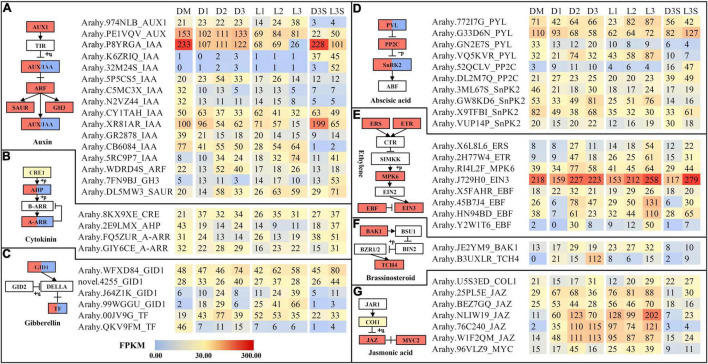
DEGs related to plant hormone signal transduction. **(A)** DEGs related to auxin signal transduction; **(B)** DEGs related to cytokinin signal transduction; **(C)** DEGs related to gibberellin signal transduction; **(D)** DEGs related to abscisic signal transduction; **(E)** DEGs related to ethylene signal transduction; **(F)** DEGs related to brassinosteroid signal transduction; **(G)** DEGs related to jasmonic acid signal transduction. Only those with FPKM value ≥ 30 in at least one sample are listed; the others can be found in Supplementary Table 9.

Plant hormones play an important role in the life of plants. Therefore, we explored plant hormone-related genes. Similar to circadian rhythm-related DEGs, hormone-related DEGs were also mostly upregulated. The largest number of DEGs identified in this study were auxin-related genes, followed by abscisic acid-related genes (Figure 5). Two *IAA* (*P8YRGA* and *XR81AR*) were significantly downregulated after the loss of mechanical stress and darkness. In the L3 sample, their expression dropped to the lowest level, by 8.96 and 6.67 times, respectively. In seeds, they were all downregulated in response to light (Figure 5A). The FPKM value of *EIN3* was upregulated by 2.38 times in the L3S vs. D3S comparison in response to light (Figure 5E). Our previous study also showed that *EIN3* was significantly expressed during pod development (Cui et al., 2022). Three *JAZ* (*NLIW19*, *76C240*, and *W1F2QM*) were significantly upregulated after the loss of mechanical stress and darkness. *NLIW19* expression was upregulated up to 18.36 times in the L3 vs. DM comparison (Figure 5G).

### Weighted Gene Co-expression Network Analysis

**FIGURE 6:**
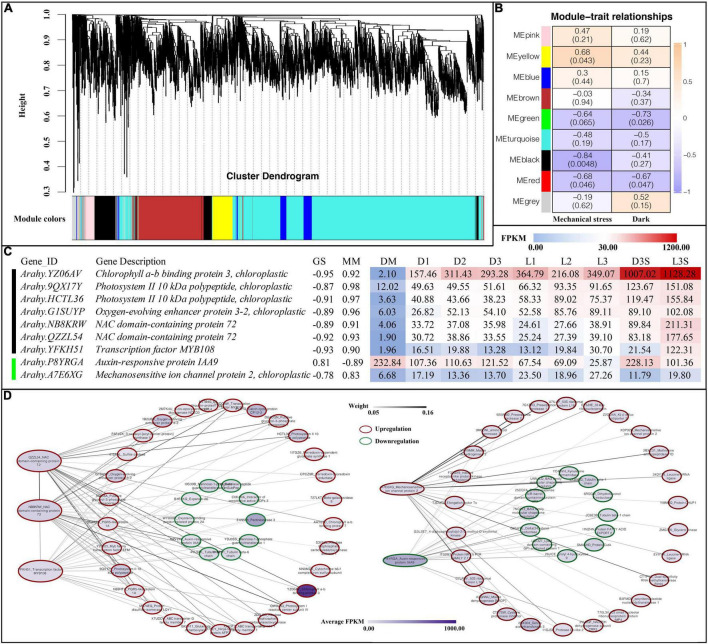
Weighted gene co-expression network analysis. **(A)** Cluster dendrogram for module construction; **(B)** module-trait relationships inside the cell; the number above represents the correlation coefficient, and the number in parentheses below represents significance; **(C)** hub gene of black and green modules, GS, gene significance; MM, module membership; **(D)** co-expression network of hub genes.

To further investigate the relationship of genes with darkness and mechanical stress, WGCNA was performed to construct the co-expression networks. A total of nine co-expression modules with 8,000 genes were identified based on their similar expression patterns (Figure 6A and Supplementary Table 10). The heatmap of module-trait correlations indicated that the black and red modules, with 377 and 386 genes, respectively, were significantly negatively correlated with mechanical stress. The yellow module, with 756 genes, was significantly positively correlated with mechanical stress. The green module (including 400 genes) and the red module were significantly negatively correlated with darkness (Figure 6B).

After screening the genes in the above modules by MM and GS, we found the following genes in the black and green modules (Figure 6B): four photosystem-related genes (one *chlorophyll a-b binding protein 3*, two *photosystems II 10 kDa polypeptide*, and one *oxygen-evolving enhancer protein 3-2*), two *NAC transcription factors 72*, one *transcription factor MYB108*, one auxin-responsive protein IAA9, and one *mechanosensitive ion channel protein 2* (Figure 6C).

In *Arabidopsis*, NAC 72 is primarily involved in reprogramming during senescence, and MYB108 acts as a negative regulator of abscisic acid-induced cell death (Kamranfar et al., 2018). Both *NAC 72* and one *MYB108* were upregulated in the pod shell after losing mechanical stress and darkness. They were all upregulated in the L3S vs. D3S comparison in response to light (Figure 6C). Co-expression network analysis showed that two *NAC 72* had a strong co-expression relationship with photosystem-related genes such as *photosystem II 10 kDa polypeptide* and *oxygen-evolving enhancer protein*, which were all upregulated after the loss of mechanical stress and darkness (Figure 6D). Cell wall-related genes such as *expansin-A6* and *pectinesterase 3*, *IAA8*, and two *tubulins* were also included in this co-expression network and were all downregulated after the loss of mechanical stress and darkness. This indicates that changes in the external environment transmitted signals through photosystem-related genes to transcription factors such as NAC and MYB, transmitting signals to hormones, cell walls, and cell structure-related genes to regulate pod developmental arrest.

*IAA9* (*P8YRGA*) was significantly downregulated in the pod, which could be involved in inhibiting pod growth by auxin signaling. *Mechanosensitive ion channel protein* (*A7E6XG*) was another interesting gene: it was upregulated in both D and L samples (the upregulation in L was more obvious). In addition, it was also upregulated in seed in response to light. Therefore, this gene was regulated not only by mechanical stress but also by light (Figure 6C). Co-expression network analysis demonstrated that *mechanosensitive ion channel protein* had a very strong co-expression relationship with protein modification-related genes such as *methionine aminopeptidase 1D* and *presequence protease*, which were all upregulated after the loss of mechanical stress and darkness. As such, the *mechanosensitive ion channel protein* could signal by altering protein modifications. *IAA9* (*P8YRGA*), which was downregulated, had a co-expression relationship with downregulated genes such as *protein fatty acid export 2* and *BAG family molecular chaperone regulator* (Figure 6D).

### Quantitative Real-Time Polymerase Chain Reaction Validation of Key Genes

**FIGURE 7:**
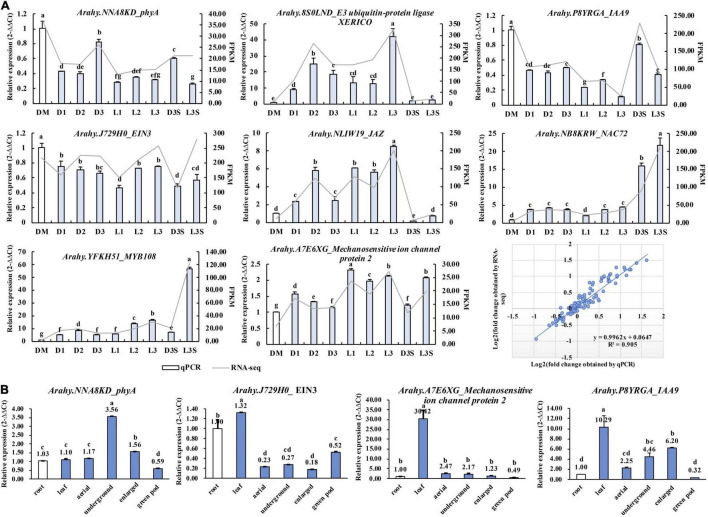
qRT-PCR validation of key genes identified in the transcriptome. **(A)** Validation of the RNA-Seq results by qRT-PCR; **(B)** qRT-PCR detecting the expression of key genes in the root, leaf, aerial peg, underground peg, enlarged pod, and exposed green pod. Different lowercase letters indicate a significant difference at *p* ≤ 0.05 (Duncan’s multiple range test).

To test the reliability of RNA-seq results, eight genes, including *phyA* (*NNA8KD*), *EIN3* (*J729H0*), *MYB108* (*YFKH51*), and *IAA9* (*P8YRGA*) were verified by qRT-PCR. The results of qRT-PCR were consistent with those of transcriptomes with high degrees of fitting (*R*^2^ = 0.905) (Figure 7A).

As the pod sticks out of the soil, it stops enlargement. This is the opposite of peg penetration and enlargement. Therefore, we used previous transcriptome data (Clevenger et al., 2016) to check whether these genes we screened also played a role in the positive process of pod penetration and enlargement (Supplementary Figure 4a). The *chlorophyll a-b binding protein 3* (*YZ06AV*), *IAA9* (*P8YRGA*), *phyA* (*NNA8KD*), and *mechanosensitive ion channel protein* (*A7E6XG*) showed an expression pattern different from that of the excavation. The upregulated/downregulated genes during peg penetration and enlargement were downregulated/upregulated after the loss of mechanical stress and darkness. The two *E3 ubiquitin ligases* and one *calcium-binding protein* were upregulated in both forward and reverse excavation, indicating that they could regulate the initiation or arrest of pod development at the non-transcriptional level (Supplementary Figure 4a).

We also verified the expression of *phyA* (*NNA8KD*), *EIN3* (*J729H0*), *IAA9* (*P8YRGA*), and *mechanosensitive ion channel protein* (*A7E6XG*) in the root, leaf, aerial peg, underground peg, and enlarged pod. We exposed green pods through qRT-PCR (Figure 7B), while *phyA* and *IAA9* were upregulated along with peg penetration and enlargement. In the illuminated green pod, their expression was significantly downregulated, which is consistent with the transcriptome results. The *mechanosensitive ion channel protein* was downregulated along with peg penetration and enlargement. This suggests that these three genes play an important role in developing peanut pods at the transcriptional level (Figure 7B).

## Discussion

**FIGURE 8:**
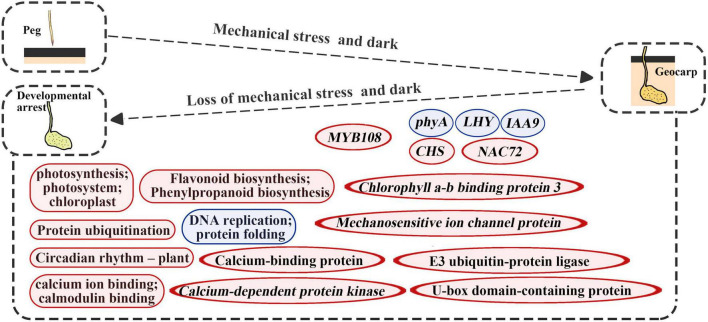
Diagrams summarize changes in key molecular levels of developmental arrest caused by the absence of dark and mechanical stress. Round rectangles represent the GO term or KEGG pathways and the ellipse represents the gene; red represents upregulation and blue represents downregulation.

In this study, gene expression changes were detected during the reverse process of geocarpy in peanuts. Along with the results of both forward and reverse processes, this revealed the importance of some genes. For example, the *phyA* was again identified as an important gene in pod development since its expression increased during the forward process and decreased during the reverse process. In addition, *IAA9* and *mechanosensitive ion channel protein* were first found to regulate pod enlargement at the transcriptional level (Figure 8).

### Differentially Expressed Gene-Enriched Pathways After the Loss of Mechanical Stress and Darkness

Developmental arrest after pod excavation can be explained by the significant downregulation of DEGs related to DNA replication, ribosomes, and protein folding (Tables 1, 2). Genes related to photosynthesis played an important part in pod development (Kumar et al., 2019). Upregulated DEGs were enriched in protein ubiquitination and flavonoid biosynthesis (Figure 8). Protein ubiquitination has always been closely related to photophogenesis. COP1 (Constitutive Photomorphogenic 1), as a star factor, is an E3 ubiquitin ligase in photophogenesis (Osterlund et al., 2000). Previous research has also confirmed that protein ubiquitination plays an important role in peanut pod development (Zhu et al., 2014; Gao et al., 2017; Liu et al., 2019). This study’s main upregulated ubiquitination-related genes were E*3 ubiquitin-protein ligase* and *U-box domain-containing protein*, which could function at the non-transcriptional level (Figures 4, 8). CHS, a key enzyme in flavonoid biosynthesis, was massively upregulated in this study (Figures 3, 8). CHS was also involved in lignin synthesis (Eloy et al., 2017). Therefore, upregulated CHS expression was consistent with the significantly increased lignification of the excavated pods (Zhu et al., 2013). Previous studies have demonstrated that lignification is a mechanism of disease resistance (Vance et al., 1980). However, subsequent studies have shown that lignin has different functions for different types of plant cells (Barros et al., 2015). Thus, in geocarpy, it is unclear whether the lignin change resists stress or has other functions.

### *Phy A, AUX/IAA*, and *Mechanosensitive Ion Channel Protein* Genes in Peanut Pod Development

*Phy A* has been identified multiple times as a vital candidate for peanut pod development (Thompson et al., 1992; Xia et al., 2013; Liu et al., 2020; Zhang et al., 2016). Light is one of the most important factors regulating plant growth and development. *Phy A* is the far-red light receptor, which translocates from the cytosol/nucleus into the nucleus/cytosol to trigger physiological responses (Sheerin and Hiltbrunner, 2017). The transcription levels of phyA increase with peg penetration, which requires further research. This study identified several auxin-related DEGs, of which *AUX/IAA* genes were the most abundant, and *IAA9* was identified as the hub gene during pod development (Figure 6). In *Arabidopsis*, studies have shown that AUX/IAA links light perception and auxin response pathways (Reed, 2001; Liscum and Reed, 2002). Evidence suggests that at least seven different AUX/IAAs interact with phyA, and phyA can phosphorylate several AUX/IAA proteins *in vitro* (Soh et al., 1999; Colón-Carmona et al., 2000).

As an important plant hormone, auxin participates in plant growth and development. Changes to and redistribution of auxin were also involved in developing peanut pods (Moctezuma, 2003). In addition, AUX/IAA proteins are associated with gravitropism. Both IAA8 and IAA17 were involved in root gravitropism of *Arabidopsis* (Leyser et al., 1996; Rouse et al., 1998; Arase et al., 2012). There are complex interrelationships between gravitropism and light (Li et al., 2020; Yang et al., 2020). Therefore, we hypothesized that AUX/IAA was most likely involved in regulating peg geotropism and dark morphogenesis during geocarpy, though the specific mechanism requires further study. The expression level of *IAA9* significantly increased in peg penetration and decreased after excavation. Promoter analysis showed seven light response elements and one rhythm response element on the promoter of *IAA9*, of which the elements close to ATG were mainly light responses (Supplementary Figure 4b). Therefore, *IAA9* was most likely regulated by light at the transcriptional level.

*Mechanosensitive ion channel protein* was another interesting gene identified in this study (Figure 8). Piezo is the most important mechanosensor in mammals, and in *Arabidopsis*, AtPiezo responds to mechanical stimuli at the transcriptional level (Coste et al., 2012; Mousavi et al., 2021). In our previous study, the homologous proteins of piezo were identified in peanuts. However, their transcriptional level did not change as mechanical stress changed. Therefore, the discovery of the *mechanosensitive ion channel protein* and its change of transcription level in this study were interesting. In plants, mechanosensitive ion channels are a common mechanism to perceive and respond to mechanical force, transducing membrane tension directly into ion flux. There are three families of mechanosensitive ion channels that have been identified in plants: the MscS-like (MSL), Mid1-complementing activity (MCA), and two-pore potassium (TPK) families (Hamilton et al., 2015). Molecular genetic approaches in *Arabidopsis thaliana* and other model plants have recently added another dimension to the study of MS ion channels. However, while research on mechanosensitive ion channels in *Arabidopsis* and other model plants has increased our understanding of these processes (Basu et al., 2020; Tran et al., 2021), much remains unknown, especially in non-model plants such as peanuts.

### Possible Signaling Mechanism Involved in the Regulation of Pod Development

Phosphorylation is a universal regulatory mechanism in plant growth and development and transmits signals and regulates life activities by regulating protein stability, protein interaction, and protein-DNA interaction. The highest concentration of plant phosphoproteins is found in the chloroplast (Bennett, 1991). In addition to transcriptional and translational differences, plant circadian signal transduction networks contain many post-translational mechanisms, of which phosphorylation is one of the most important mechanisms (Kusakina and Dodd, 2012). Moreover, phytochrome has long been considered an autophosphorylated serine/threonine-protein kinase, and its autophosphorylation has been reported to play an important role in regulating plant light signals (Hoang et al., 2019). Biological and abiotic stresses in plants change the concentration of cytoplasmic calcium, which activates calcium-regulated protein kinases and transmits signals through phosphorylation mechanisms (Saito and Uozumi, 2020). Therefore, in this study, photochromes and circadian rhythms were considered to play an important role in pod development (Figures 3, 8). In addition, a large number of chloroplasts, calmodulin-related genes, and numerous protein ubiquitination-related genes were found in DEGs (Supplementary Table 3). As such, we hypothesized that at least two signal transduction pathways were involved in pod development: one starts at chloroplasts and transmits signals downstream through phosphorylation; the other transmits signals downstream through calcium signaling, phosphorylation, and ubiquitination from abiotic stress.

## Conclusion

Developmental stagnation occurs in peanut pods after the loss of mechanical stress and darkness, and chloroplast, photosynthesis, protein ubiquitination, circadian rhythm, flavonoid biosynthesis, and calmodulin-related genes are all significantly upregulated, while gene-related DNA replication, ribosome, and protein folding were all significantly downregulated. Combining peg penetration and excavation, *phyA*, *IAA9*, and *mechanosensitive ion channel protein* genes were identified as key genes that could be involved in pod development. In addition, we hypothesized that two signaling pathways, beginning with chloroplast and calcium signaling, played important roles in peanut pod development. Our results provide useful information for the development of peanut pods and geocarpy.

## Data Availability Statement

The raw data presented in the study are deposited in the SRA repository, accession number: PRJNA770556. The raw data has been released before submission.

## Author Contributions

XL, XD, and YC conceived this study. XL and XD supervised the research. YC performed the experiments. YC, XL, JB, and XD analyzed the data. All authors prepared, read, and approved the manuscript for publication.

## Conflict of Interest

The authors declare that the research was conducted in the absence of any commercial or financial relationships that could be construed as a potential conflict of interest.

## Publisher’s Note

All claims expressed in this article are solely those of the authors and do not necessarily represent those of their affiliated organizations, or those of the publisher, the editors and the reviewers. Any product that may be evaluated in this article, or claim that may be made by its manufacturer, is not guaranteed or endorsed by the publisher.

## Footnotes

1
https://bioinfo.ut.ee/primer3/

## Supplementary Material

Supplementary Figure 1 Illustrations for experimental materials and treatment. **(a)** Pod that stops developing after exposure; **(b)** specific materials and treatment in this study.

Supplementary Figure 2 Sample correlation analysis.

Supplementary Figure 3 The expression changes of *U-box domain-containing protein/E3 ubiquitin ligase* (*J6YH7R* and *CT4JMD*) and *EBF* (*EIN3-binding F-box protein*: *Y2W1T6*).

Supplementary Figure 4 The information of key genes involved in geocarpy. **(a)** Expression changes of key genes during peg penetration [on the right, the data were obtained from the developmental transcriptome of Tifrunner contributed by Clevenger et al. (2016)] and excavation (on the left) and **(b)**, promoter analysis of *IAA9*.

Supplementary Table 1 Primers used in qPCR.

Supplementary Table 2 Information about all DEGs, including FPKM value, chromosome location, and gene length.

Supplementary Table 3 Significant GO terms for DEGs in D vs. DM, L vs. DM, and L vs. D. The “GeneRatio” means the ratio of the number of DEGs annotated in the GO term to the total number of DEGs that can be annotated in the GO database. The “BgRatio” means the ratio of the number of background genes annotated in the GO term to the total number of background genes that can be annotated in the GO database.

Supplementary Table 4 Significant GO terms and KEGG pathways for upregulated or downregulated genes in D vs. DM. The “GeneRatio” means the ratio of the number of DEGs annotated in the GO term/KEGG pathway to the total number of DEGs that can be annotated in the GO/KEGG database. The “BgRatio” means the ratio of the number of background genes annotated in the GO term/KEGG pathway to the total number of background genes that can be annotated in the GO/KEGG database.

Supplementary Table 5 Significant GO terms and KEGG pathways for upregulated or downregulated genes in the L vs. DM comparison. The “GeneRatio” means the ratio of the number of DEGs annotated in the GO term/KEGG pathway to the total number of DEGs that can be annotated in the GO/KEGG database. The “BgRatio” means the ratio of the number of background genes annotated in the GO term/KEGG pathway to the total number of background genes that can be annotated in the GO/KEGG database.

Supplementary Table 6 Significant GO terms and KEGG pathways for upregulated or downregulated genes in L vs. D. The “GeneRatio” means the ratio of the number of DEGs annotated in the GO term/KEGG pathway to the total number of DEGs that can be annotated in the GO/KEGG database. The “BgRatio” means the ratio of the number of background genes annotated in the GO term/KEGG pathway to the total number of background genes that can be annotated in the GO/KEGG database.

Supplementary Table 7 The DEGs enriched in circadian rhythm.

Supplementary Table 8 The calmodulin-related DEGs identified in this study.

Supplementary Table 9 The DEGs enriched in plant hormone signal transduction.

Supplementary Table 10 The relationship between genes and modules. MM: module membership, the closer the value is to 1 or −1, the gene is strongly associated with the module (positive or negative).

## References

[B1] Arase F. Nishitani H. Egusa M. Nishimoto N. Sakurai S. Sakamoto N. (2012). IAA8 involved in lateral root formation interacts with the TIR1 Auxin Receptor and ARF Transcription Factors in *Arabidopsis*. *PLoS One* 7:e43414. 10.1371/journal.pone.0043414 22912871 PMC3422273

[B2] Barros J. Serk H. Granlund I. Pesquet E. (2015). The cell biology of lignification in higher plants. *Ann. Bot.* 115 1053–1074. 10.1093/aob/mcv046 25878140 PMC4648457

[B3] Basu D. Shoots J. M. Haswell E. S. (2020). Interactions between the N-and C-termini of the mechanosensitive ion channel At MSL10 are consistent with a three-step mechanism for activation. *J. Exp. Bot.* 71 4020–4032. 10.1093/jxb/eraa192 32280992

[B4] Bennett J. (1991). Protein phosphorylation in green plant chloroplasts. *Annu. Rev. Plant Biol.* 42 281–311. 10.1146/annurev.pp.42.060191.001433

[B5] Bertioli D. J. Jenkins J. Clevenger J. Dudchenko O. Gao D. Seijo G. (2019). The genome sequence of segmental allotetraploid peanut *Arachis hypogaea*. *Nat. Genet.* 51 877–884. 10.1038/s41588-019-0405-z 31043755

[B6] Chen C. Chen H. Zhang Y. Thomas H. R. Frank M. H. He Y. (2020). TBtools: an integrative toolkit developed for interactive analyses of big biological data. *Mol. Plant* 13 1194–1202. 10.1016/j.molp.2020.06.009 32585190

[B7] Chen X. Yang Q. Li H. Li H. Hong Y. Pan L. (2016). Transcriptome-wide sequencing provides insights into geocarpy in peanut (*Arachis hypogaea* L.). *Plant Biotechnol. J.* 14 1215–1224. 10.1111/pbi.12487 26502832 PMC11388922

[B8] Chen X. Zhu W. Azam S. Li H. Zhu F. Li H. (2013). Deep sequencing analysis of the transcriptomes of peanut aerial and subterranean young pods identifies candidate genes related to early embryo abortion. *Plant Biotechnol. J.* 11 115–127. 10.1111/pbi.12018 23130888

[B9] Clevenger J. Chu Y. Scheffler B. Ozias-Akins P. (2016). A developmental transcriptome map for allotetraploid *Arachis hypogaea*. *Front. Plant Sci.* 7:1446. 10.3389/fpls.2016.01446 27746793 PMC5043296

[B10] Colón-Carmona A. Chen D. L. Yeh K. C. Abel S. (2000). Aux/IAA proteins are phosphorylated by phytochrome in vitro. *Plant Physiol.* 124 1728–1738. 10.1104/pp.124.4.1728 11115889 PMC59870

[B11] Coste B. Xiao B. Santos J. S. Syeda R. Grandl J. Spencer K. S. (2012). Piezo proteins are pore-forming subunits of mechanically activated channels. *Nature* 483 176–181. 10.1038/nature10812 22343900 PMC3297710

[B12] Cui Y. Bian J. Guan Y. Xu F. Han X. Deng X. (2022). Genome-Wide analysis and expression profiles of ethylene signal genes and AP2/ERFs in Peanut (*Arachis hypogaea* L.). *Front. Plant Sci.* 13:828482. 10.3389/fpls.2022.828482 35371146 PMC8968948

[B13] Cui Y. Zhai Y. Flaishman M. Li J. Chen S. Zheng C. (2021). Ethephon induces coordinated ripening acceleration and divergent coloration responses in fig (*Ficus carica* L.) flowers and receptacles. *Plant Mol. Biol.* 105 347–364. 10.1007/s11103-020-01092-x 33185823

[B14] Eloy N. B. Voorend W. Lan W. Saleme M. D. L. S. Cesarino I. Vanholme R. (2017). Silencing CHALCONE SYNTHASE in maize impedes the incorporation of tricin into lignin and increases lignin content. *Plant Physiol.* 173 998–1016. 10.1104/pp.16.01108 27940492 PMC5291018

[B15] Gao C. Wang P. Zhao S. Zhao C. Xia H. Hou L. (2017). Small RNA profiling and degradome analysis reveal regulation of microRNA in peanut embryogenesis and early pod development. *BMC Genomics* 18:220. 10.1186/s12864-017-3587-8 28253861 PMC5335773

[B16] Hamilton E. S. Schlegel A. M. Haswell E. S. (2015). United in diversity: mechanosensitive ion channels in plants. *Annu. Rev. Plant Biol.* 66 113–137. 10.1146/annurev-arplant-043014-114700 25494462 PMC4470482

[B17] Han X. Huang X. Deng X. W. (2020). The photomorphogenic central repressor COP1: conservation and functional diversification during evolution. *Plant Commun.* 1:100044. 10.1016/j.xplc.2020.100044 33367240 PMC7748024

[B18] Hoang Q. T. Han Y. J. Kim J. I. (2019). Plant phytochromes and their phosphorylation. *Int. J. Mol. Sci.* 20:3450. 10.3390/ijms20143450 31337079 PMC6678601

[B19] Kamranfar I. Xue G. P. Tohge T. Sedaghatmehr M. Fernie A. R. Balazadeh S. (2018). Transcription factor RD 26 is a key regulator of metabolic reprogramming during dark-induced senescence. *New Phytol.* 218 1543–1557. 10.1111/nph.15127 29659022

[B20] Kim S. J. Ryu M. Y. Kim W. T. (2012). Suppression of *Arabidopsis* RING-DUF1117 E3 ubiquitin ligases, AtRDUF1 and AtRDUF2, reduces tolerance to ABA-mediated drought stress. *Biochem. Biophys. Res. Commun.* 420 141–147. 10.1016/j.bbrc.2012.02.131 22405823

[B21] Ko J. H. Yang S. H. Han K. H. (2006). Upregulation of an *Arabidopsis* RING-H2 gene, XERICO, confers drought tolerance through increased abscisic acid biosynthesis. *Plant J.* 47 343–355. 10.1111/j.1365-313x.2006 16792696

[B22] Kohl M. Wiese S. Warscheid B. (2011). “Cytoscape: software for visualization and analysis of biological networks,” in *Data Mining in Proteomics*, eds Hamacher M. Eisenacher M. Stephan C. (Totowa, NJ: Humana Press), 291–303. 10.1007/978-1-60761-987-1_18 21063955

[B23] Kumar R. Pandey M. K. Roychoudhry S. Nayyar H. Kepinski S. Varshney R. K. (2019). Peg biology: deciphering the molecular regulations involved during peanut peg development. *Front. Plant Sci.* 10:1289. 10.3389/fpls.2019.01289 31681383 PMC6813228

[B24] Kusakina J. Dodd A. N. (2012). Phosphorylation in the plant circadian system. *Trends Plant Sci.* 17 575–583. 10.1016/j.tplants.2012.06.008 22784827

[B25] Langfelder P. Horvath S. (2008). WGCNA: an R package for weighted correlation network analysis. *BMC Bioinform.* 9:559. 10.1186/1471-2105-9-559 19114008 PMC2631488

[B26] Leyser H. O. Pickett F. B. Dharmasiri S. Estelle M. (1996). Mutations in the AXR3 gene of *Arabidopsis* result in altered auxin response including ectopic expression from the SAUR-AC1 promoter. *Plant J.* 10 403–413. 10.1046/j.1365-313x.1996 8811856

[B27] Li F. L. (2020). *Study on Mechanism of Light Signal and BR Regulating the Development of Peanut Pod [D].* jinan: Shandong University, 10.27272/d.cnki.gshdu.2020.000623

[B28] Li Y. Yuan W. Li L. Miao R. Dai H. Zhang J. (2020). Light-dark modulates root hydrotropism associated with gravitropism by involving amyloplast response in *Arabidopsis*. *Cell Rep.* 32:108198. 10.1016/j.celrep.2020.108198 32997985

[B29] Li Z. Liu W. Wang Q. Zhang G. (2014). Effects of dark and mechanical stimulation on phytohormones content in peanut gynophore. *Chinese J. Oil Crop Sci.* 6 784–788.

[B30] Liscum E. Reed J. W. (2002). Genetics of Aux/IAA and ARF action in plant growth and development. *Plant Mol. Biol.* 49 387–400. 10.1023/a:1015255030047 12036262

[B31] Liu H. Liang X. Lu Q. Li H. Liu H. Li S. (2020). Global transcriptome analysis of subterranean pod and seed in peanut (*Arachis hypogaea* L.) unravels the complexity of fruit development under dark condition. *Sci. Rep.* 10:13050. 10.1038/s41598-020-69943-7 32747681 PMC7398922

[B32] Liu Y. Zhu J. Sun S. Cui F. Han Y. Peng Z. (2019). Defining the function of SUMO system in pod development and abiotic stresses in Peanut. *BMC Plant Biol.* 19:593. 10.1186/s12870-019-2136-9 31884953 PMC7194008

[B33] Livak K. J. Schmittgen T. D. (2001). Analysis of relative gene expression data using real-time quantitative PCR and the 2−ΔΔCT method. *Methods* 25 402–408. 10.1006/meth.2001.1262 11846609

[B34] Moctezuma E. (2003). The peanut gynophore: a developmental and physiological perspective. *Canad. J. Bot.* 81 183–190. 10.1186/s12870-019-2136-9 31884953 PMC7194008

[B35] Mousavi S. A. Dubin A. E. Zeng W. Z. Coombs A. M. Do K. Ghadiri D. A. (2021). PIEZO ion channel is required for root mechanotransduction in *Arabidopsis thaliana*. *Proc. Natl. Acad. Sci. U.S.A.* 118:e2102188118. 10.1073/pnas.2102188118 33975957 PMC8158017

[B36] Osterlund M. T. Hardtke C. S. Wei N. Deng X. W. (2000). Targeted destabilization of HY5 during light-regulated development of *Arabidopsis*. *Nature* 405 462–466. 10.1038/35013076 10839542

[B37] Pan R. Z. (1982). Research progress on the mechanism of peanut podding into the ground. *Peanut Sci. Technol.* 4, 1–4.

[B38] Pattee H. E. Johns E. B. Singleton J. A. Sanders T. H. (1974). Composition changes of peanut fruit parts during maturation. *Peanut. Sci.* 1 57–62. 10.3146/i0095-3679-1-2-6

[B39] Reed J. W. (2001). Roles and activities of Aux/IAA proteins in *Arabidopsis*. *Trends Plant Sci.* 6 420–425. 10.1016/s1360-1385(01)02042-8 11544131

[B40] Rouse D. Mackay P. Stirnberg P. Estelle M. Leyser O. (1998). Changes in auxin response from mutations in an AUX/IAA gene. *Science* 279 1371–1373. 10.1126/science.279.5355.1371 9478901

[B41] Saito S. Uozumi N. (2020). Calcium-regulated phosphorylation systems controlling uptake and balance of plant nutrients. *Front. Plant Sci.* 11:44. 10.3389/fpls.2020.00044 32117382 PMC7026023

[B42] Sheerin D. J. Hiltbrunner A. (2017). Molecular mechanisms and ecological function of far-red light signalling. *Plant Cell Environ.* 40 2509–2529. 10.1111/pce.12915 28102581

[B43] Shlamovitz N. Ziv M. Zamski E. (1995). Light, dark and growth regulator involvement in groundnut (*Arachis hypogaea* L.) pod development. *Plant Growth Regul.* 16 37–42. 10.1007/BF00040505

[B44] Soh M. S. Hong S. H. Kim B. C. Vizir I. Park D. H. Choi G. (1999). Regulation of both light- and auxin-mediated development by the *Arabidopsis* IAA3/SHY2 gene. *J. Plant Biol.* 42:239. 10.1007/BF03030485

[B45] Tan D. Zhang Y. Wang A. (2010). A review of geocarpy and amphicarpy in angiosperms, with special reference to their ecological adaptive significance. *Chin. J. Plant Eco.* 34 72–88. 10.3773/j.issn.1005-264x.2010.01.011

[B46] Thompson L. K. Burgess C. L. Skinner E. N. (1992). Localization of phytochrome during peanut (*Arachis hypogaea*) gynophore and ovule development. *Am. J. Bot.* 79 828–832. 10.1002/j.1537-2197

[B47] Tran D. Girault T. Guichard M. Thomine S. Leblanc-Fournier N. Moulia B. (2021). Cellular transduction of mechanical oscillations in plants by the plasma-membrane mechanosensitive channel MSL10. *Proc. Natl. Acad. Sci. U.S.A.* 118:e1919402118. 10.1073/pnas.1919402118 33372153 PMC7817121

[B48] Vance C. P. Kirk T. K. Sherwood R. T. (1980). Lignification as a mechanism of disease resistance. *Annu. Rev. Phytopathol.* 18 259–288. 10.1146/annurev.py.18.090180.001355

[B49] Wang J. Qi F. Zheng Z. Sun Z. Tian M. Wang X. (2021). Global transcriptome analyses provide into several fatty acid biosynthesis-related genes in peanut (*Arachis hypogaea* L.). *Trop Plant Biol.* 14 267–282. 10.1007/s12042-021-09285-4

[B50] Xia H. Zhao C. Hou L. Li A. Zhao S. Bi Y. (2013). Transcriptome profiling of peanut gynophores revealed global reprogramming of gene expression during early pod development in darkness. *BMC Genomics* 14:517. 10.1186/1471-2164-14-517 23895441 PMC3765196

[B51] Yang P. Wen Q. Yu R. Han X. Deng X. W. Chen H. (2020). Light modulates the gravitropic responses through organ-specific PIFs and HY5 regulation of LAZY4 expression in *Arabidopsis*. *Proc. Natl. Acad Sci. U.S.A.* 117 18840–18848. 10.1073/pnas.2005871117 32690706 PMC7414047

[B52] Zamski E. Ziv M. (1976). Pod formation and its geotropic orientation in the peanut, *Arachis hypogaea* L., in relation to light and mechanical stress. *Ann. Bot.* 40 631–636. 10.1093/oxfordjournals.aob.a085173

[B53] Zhang M. (1986). Peanut variety-Haihua 1. *Agric. Technol. Newsl.* 16, 7, 37.

[B54] Zhang Y. Wang P. Xia H. Zhao C. Hou L. Li C. (2016). Comparative transcriptome analysis of basal and zygote-located tip regions of peanut ovaries provides insight into the mechanism of light regulation in peanut embryo and pod development. *BMC Genomics* 17:606. 10.1186/s12864-016-2857-1 27514934 PMC4982202

[B55] Zhao C. Zhao S. Hou L. Xia H. Wang J. Li C. (2015). Proteomics analysis reveals differentially activated pathways that operate in peanut gynophores at different developmental stages. *BMC Plant Biol.* 15:188. 10.1186/s12870-015-0582-6 26239120 PMC4523997

[B56] Zhu W. Chen X. Li H. Zhu F. Hong Y. Varshney R. K. (2014). Comparative transcriptome analysis of aerial and subterranean pods development provides insights into seed abortion in peanut. *Plant Mol. Biol.* 85 395–409. 10.1007/s11103-014-0193-x 24793121 PMC4152868

[B57] Zhu W. Zhang E. Li H. Chen X. Zhu F. Hong Y. (2013). Comparative proteomics analysis of developing peanut aerial and subterranean pods identifies pod swelling related proteins. *J. Proteom.* 91 172–187. 10.1016/j.jprot.2013.07.002 23851312

[B58] Zielinski R. E. (1998). Calmodulin and calmodulin-binding proteins in plants. *Annu. Rev. Plant Biol.* 49 697–725. 10.1146/annurev.arplant.49.1.697 15012251

[B59] Ziv M. Zamski E. (1975). Geotropic responses and pod development in gynophore explants of peanut (*Arachis hypogaea* L.) cultured in vitro. *Ann. Bot.* 39 579–583. 10.1093/oxfordjournals.aob.a084968

