# Supplementary figures and images for: Analysis of the Transcriptional Dynamics of Regulatory Genes During Peanut Pod Development Caused by Darkness and Mechanical Stress
Source: Front Plant Sci. 2022 May 26;13:904162. doi: 10.3389/fpls.2022.904162 (PMC9178256; doi:10.3389/fpls.2022.904162)

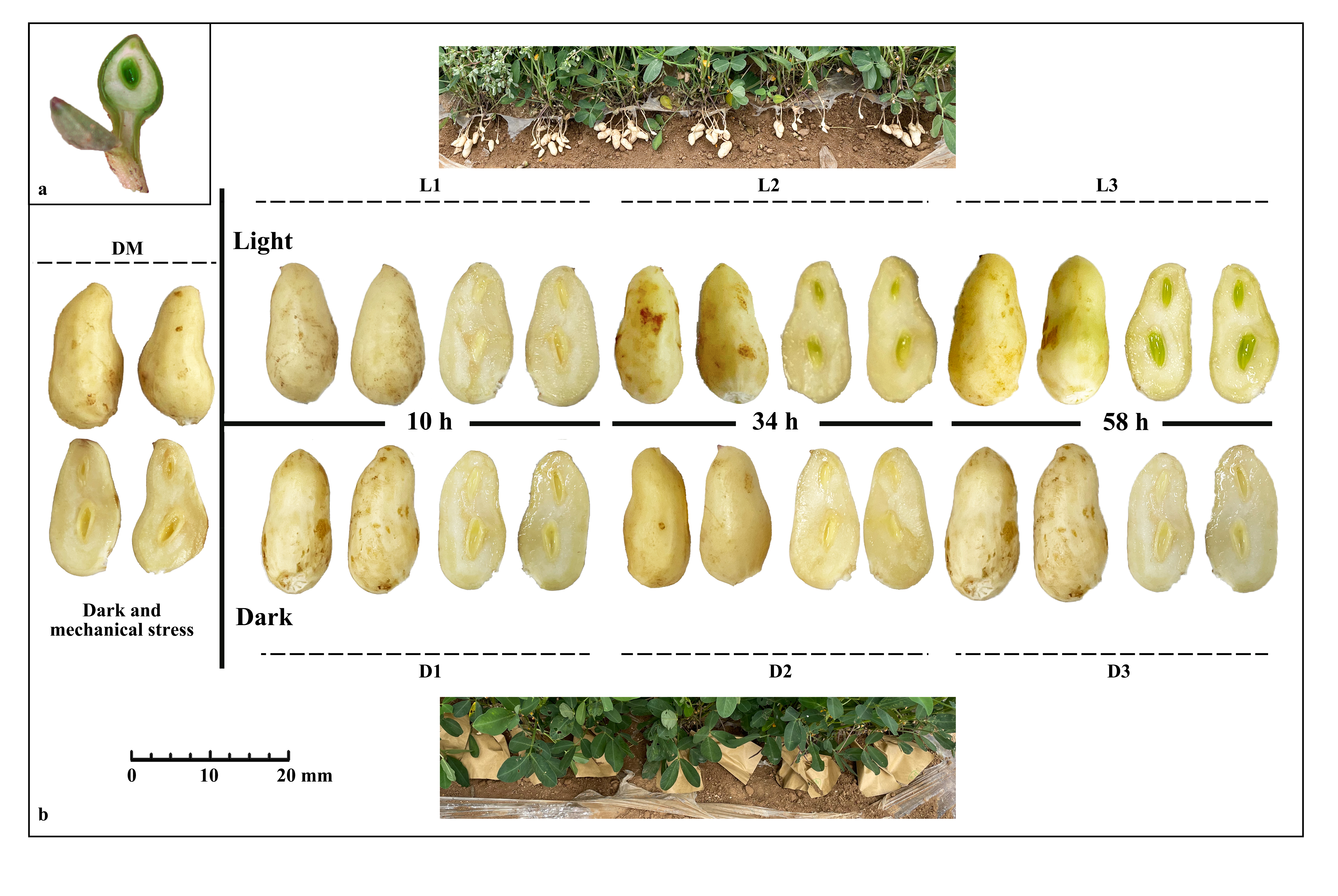

Supplement: Supplementary Figure 1 — Illustrations for experimental materials and treatment. (a) Pod that stops developing after exposure; (b) specific materials and treatment in this study. [file Image_1.JPEG]

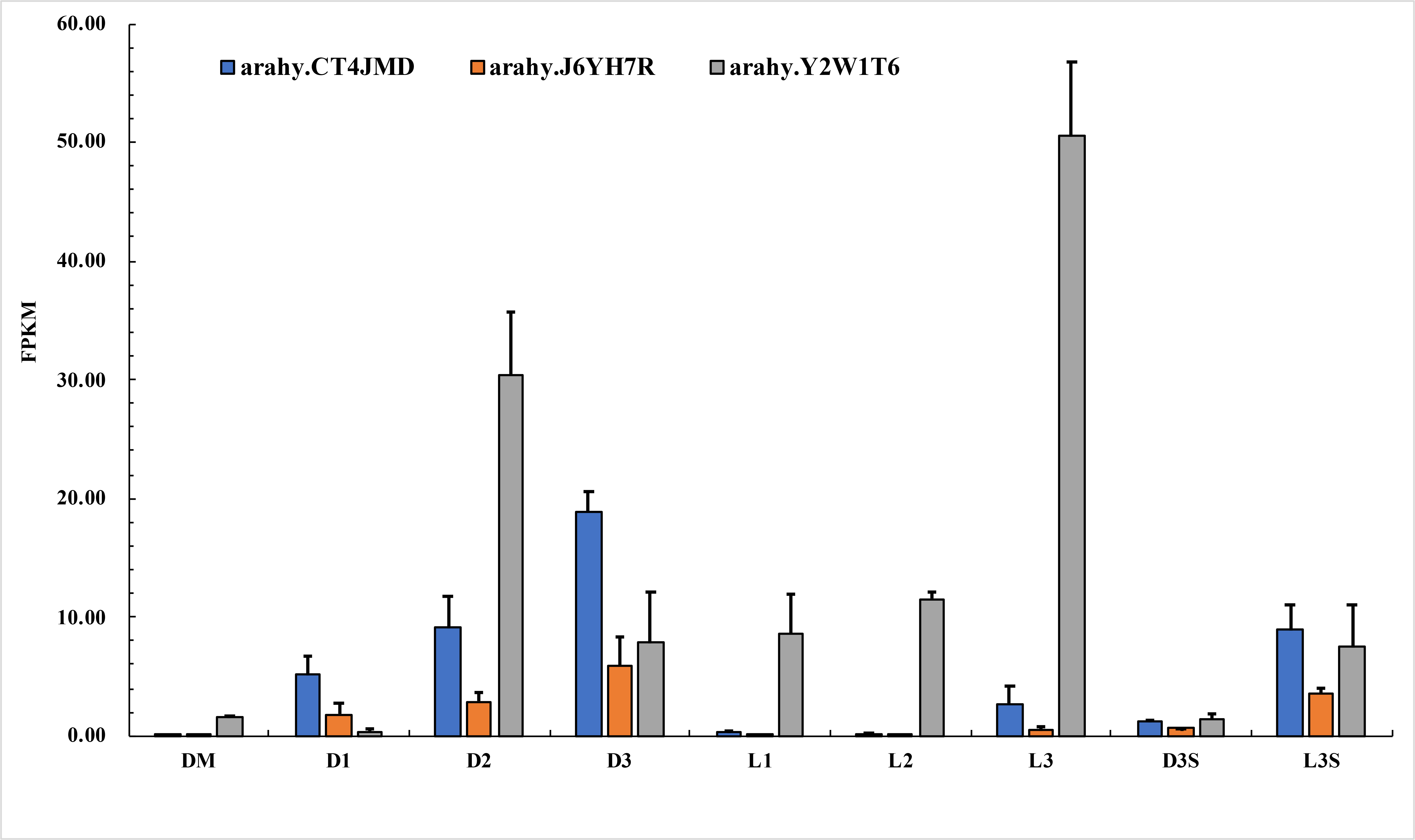

Supplement: Supplementary Figure 3 — The expression changes of U-box domain-containing protein/E3 ubiquitin ligase (J6YH7R and CT4JMD) and EBF (EIN3-binding F-box protein: Y2W1T6). [file Image_3.JPEG]

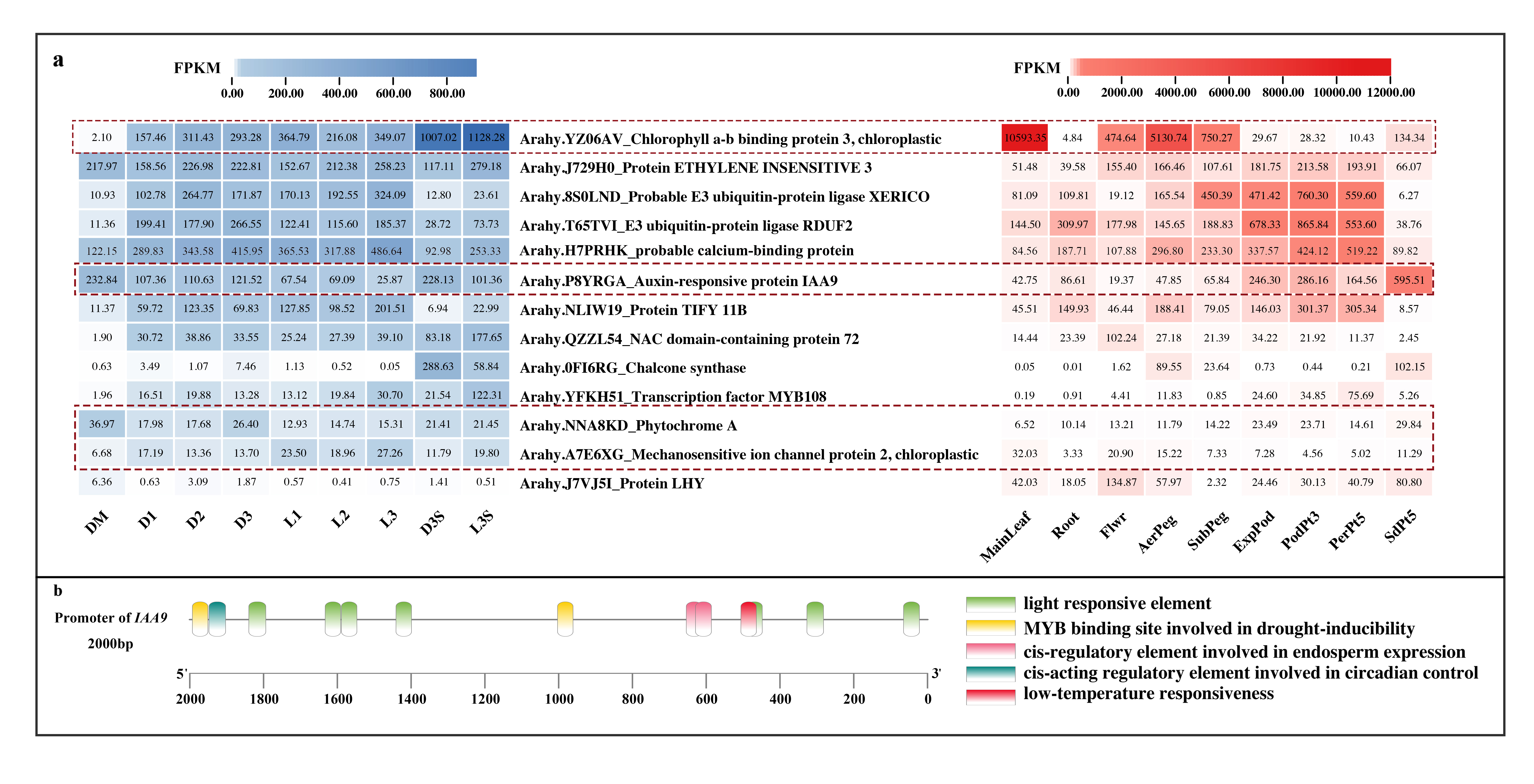

Supplement: Supplementary Figure 4 — The information of key genes involved in geocarpy. (a) Expression changes of key genes during peg penetration [on the right, the data were obtained from the developmental transcriptome of Tifrunner contributed by Clevenger et al. (2016)] and excavation (on the left) and (b), promoter analysis of IAA9. [file Image_4.JPEG]
