# Supplementary material for: Analysis of the Transcriptional Dynamics of Regulatory Genes During Peanut Pod Development Caused by Darkness and Mechanical Stress
Source: Front Plant Sci. 2022 May 26;13:904162. doi: 10.3389/fpls.2022.904162 (PMC9178256; doi:10.3389/fpls.2022.904162)
Supplement: Supplementary Table 1 — Primers used in qPCR. [file Table_1.DOCX]

**Supplementary Table 1** The primers of qPCR.

| Gene Name | Primer-F (5'-3') | Primer-R (5'-3') |
| --- | --- | --- |
| *Arahy.NNA8KD* | TCCTATGACTGCAGCAGGTG | GCCATCACCCTGTCATAACC |
| *Arahy.8S0LND* | CTTCCCTCTCCGCTATCCTC | CAGAGAGGGCAAGTCCTTTG |
| *Arahy.P8YGRA* | GCCGAGGCCTTCTTCTAACT | GACCTGAGCCTTGGTAGCTG |
| *Arahy.J729H0* | CAACCTCTCCCTTGATTGGA | TCGCGAGCTTTATCCTGATT |
| *Arahy.NLIW19* | AGACTTGCAGCCTCTTGAGC | TCTTGATGCAGGGATTTTCC |
| *Arahy.NB8KRW* | AAGAATGGAGCAATGGATCG | GACCCAGTCCGGGAAATTAT |
| *Arahy.YFKH51* | ACCCTGCACCACAAAAACTC | TGTCCCCTGTTCAAACAACA |
| *Arahy.A7E6XG* | GGTTTGGGCACAGTATTGCT | AACTGCTTCCCGATCATCAC |
| *Arahy.E3HYW*R | AAGCTTCCCTGGCAAAGCTCAA | TTCCTCAGCTGCCTTCTTATCC |
